# Acute Ulnar Shortening for Delayed Presentation of Distal Radius Growth Arrest in an Adolescent

**DOI:** 10.1155/2012/928231

**Published:** 2012-10-17

**Authors:** Prasad Ellanti, Paul Harrington

**Affiliations:** Regional Orthopaedic Unit, Our Lady's Hospital, Navan, Co-Meath, Ireland

## Abstract

Distal radius physeal fractures are common in children and adolescents. However, posttraumatic growth arrest is uncommon. The management of posttraumatic growth arrest is dependent on the severity of the deformity and the remaining growth potential of the patient. Various treatment options exist. We present a 17-year-old male with distal radius growth arrest who presented four years after the initial injury. He had a symptomatic 15 mm positive ulnar variance managed with an ulnar shortening osteotomy with the use of the AO mini distractor intraoperatively. To the best of our knowledge, an acute ulnar shortening of 15 mm is the largest reported.

## 1. Introduction

Distal radius fractures are common in children and adolescents with up to 15% of these involving the distal radial physis [1]. Posttraumatic distal radial growth arrest is uncommon despite the high incidence of physeal fractures [1]. Distal radial growth arrest can lead to a relative ulnar overgrowth leading to altered wrist mechanics, significant pain, and limitation of function [1, 2]. We report a case of an adolescent male with a painful wrist due to a significant distal radius growth arrest managed with an acute 15 mm ulnar shortening osteotomy.

## 2. Case Report

A 17-year-old right-hand-dominant adolescent male presented to our outpatient clinic with symptoms of discomfort and ulnar-sided wrist pain affecting the dominant right wrist. There was a history of a hyperextension injury to the wrist while playing Gaelic football four years previously. The family did not present for an X-ray examination at the time as it was felt that the injury was “a wrist sprain.”

Prior to presentation to our outpatient's service, the child began to complain of ulnar sided wrist pain and lately the mother had noticed a deformity with the prominence of the distal ulna.

At presentation there was radial deviation posture of the right wrist. Pronation was full, but supination was limited at 50°. Wrist flexion and extension was normal. Ulnar deviation was absent. Grip strength assessment using the Jamar dynamometer (Sammons Preston Roylan, Bolingbrook, IL, USA) showed a 50% reduction in grip strength in comparison to the contralateral normal side. There was a positive ulnar ballottement test and a positive ulnar impaction test.

**Figure 1:**
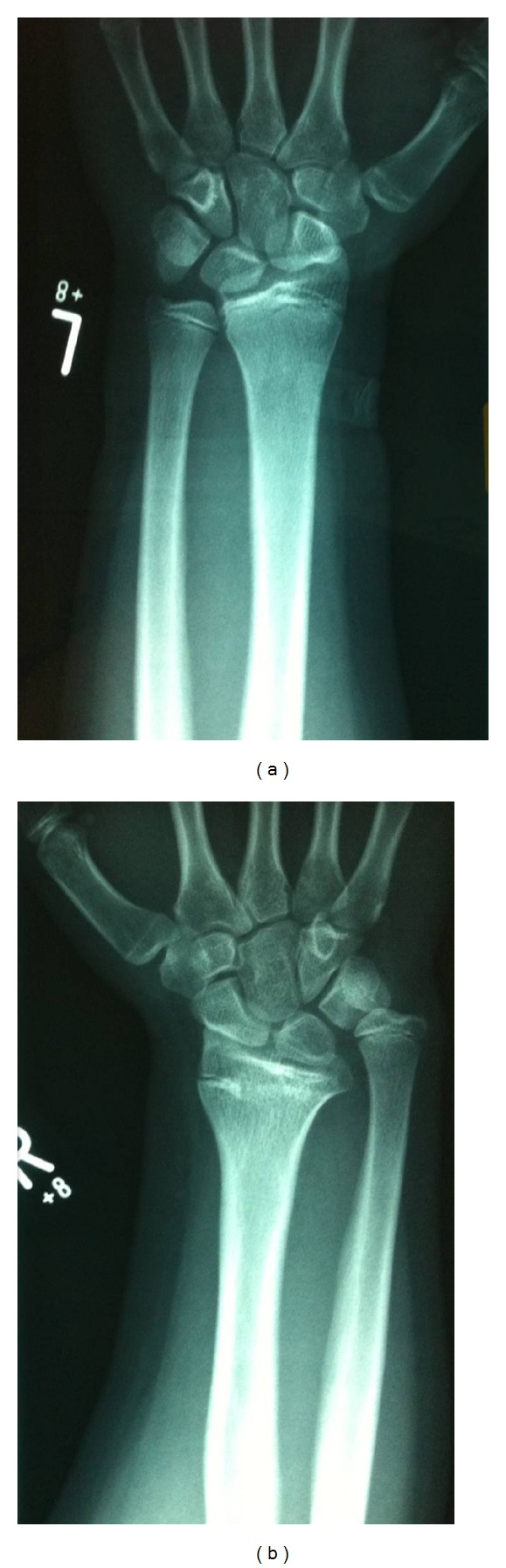
Bilateral anteroposterior radiographs of the distal radius demonstrating distal radius growth arrest on the right side compared to the normal left side.

Radiographs showed a 15 mm positive ulnar variance (Figure 1). A CT scan of the wrist was undertaken to further assess the architecture of the sigmoid notch of the distal radius. This appeared to be congruent despite the physeal growth arrest. A Modified Mayo Wrist Score [1] of 60 rated his wrist function as poor. After discussion with the patient and family, it was decided to undertake a shortening osteotomy of the ulna.

**Figure 2:**
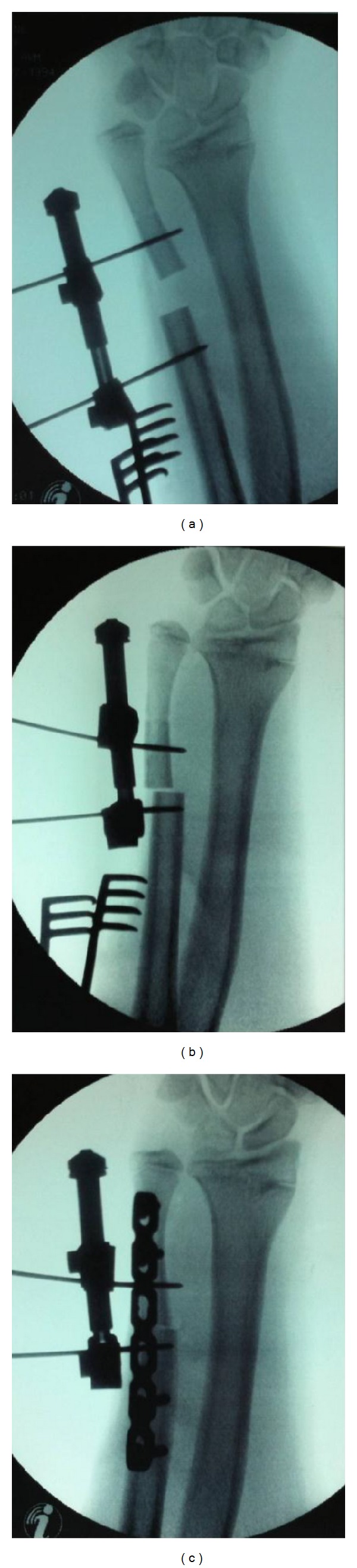
Intraoperative screening radiographs demonstrating (a) ulnar osteotomy with external fixator in situ, (b) reduction of the osteotomy bone ends, note the deformation of the Kirschner wires, (c) fixation of the osteotomy with a plate.

**Figure 3:**
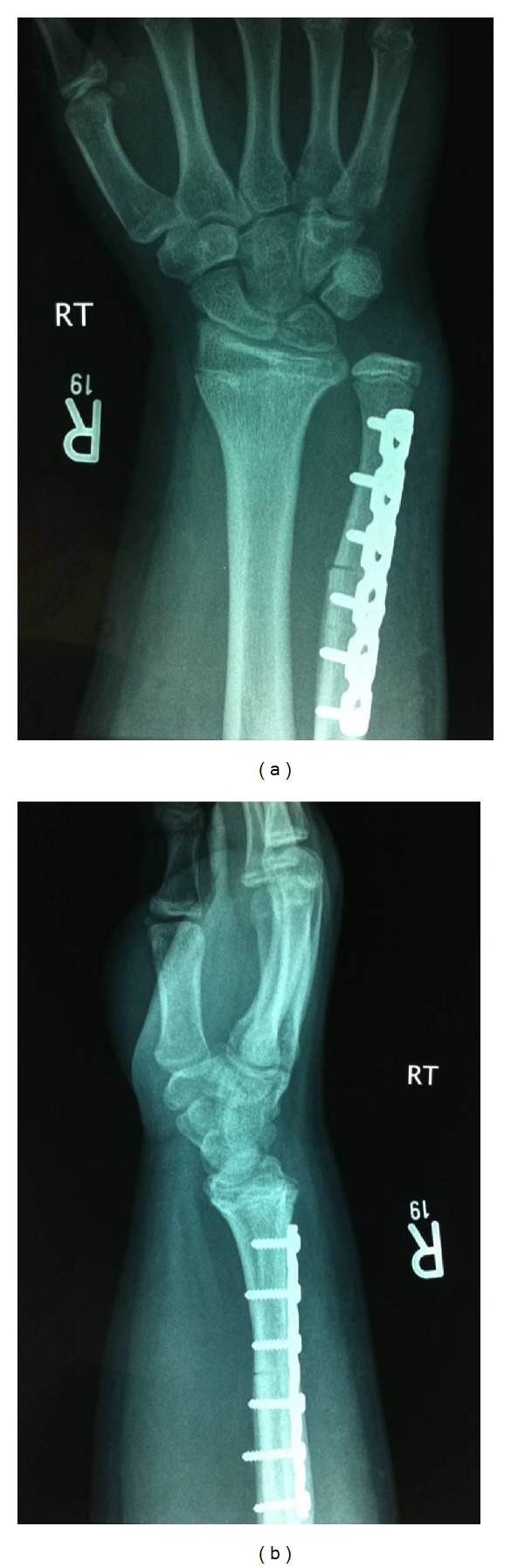
Postoperative anteroposterior and lateral radiographs demonstrating satisfactory ulnar shortening and fixation of the osteotomy site.

Surgery was performed under general anaesthesia. A direct ulnar approach to the distal ulna was performed. An excision of 15 mm from the distal ulna was planned. Either side of the osteotomy site the bone was marked for control of rotation. An AO mini distractor was used to transport the distal segment and compress the osteotomy site (Figure 2). The osteotomy was fixed and held with a 6-hole dynamic compression plate (Figure 3). The forearm was supported in a plaster of paris backslab for two weeks, at which point physiotherapy was commenced. At nine months postoperatively, range of motion was normal and pain free except for supination which was limited to 60°. Grip strength had improved to 80%. Wrist function was rated as good with a Modified Mayo Wrist Score of 90. Radiographs confirmed satisfactory union at the osteotomy site. 

## 3. Discussion

Distal physeal fractures are very common among children and adolescents; however, posttraumatic growth arrest is uncommon [1]. All types of physeal fractures have been reported to cause growth arrest. Salter-Harris type-2 fractures predominate, accounting for 75% of all physeal fractures. Growth disturbances are more common in the upper limb than the lower limb and generally carry a good prognosis. As Salter-Harris type-4 injuries cross the metaphysis, physis, and epiphysis, they carry a greater potential for growth arrest. In the Salter-Harris type-5 injuries as no fracture is evident on the initial radiographs, the diagnosis is often made retrospectively as clinical or radiographic deformity develops. This is the likely cause in our patient.

There are several methods to correct the relative ulnar “overgrowth.” Ulnar shortening osteotomy is used in the skeletally mature or those with little remaining growth potential. Ulnar epiphysiodesis on its own or with a shortening osteotomy is useful in those who are skeletally immature. Various lengthening osteotomies of the radius have been described [1, 2].

As posttraumatic growth arrest is uncommon, there is a relative paucity in the literature regarding its management. Waters et al. [1] published a large series involving 30 children and adolescents treated surgically for posttraumatic distal radius growth arrest with an average age of 14.8 years (12.3–20 years) at the time of corrective surgery. Eighteen of these patients underwent ulnar shortening osteotomy (mean 4.5 mm). The largest positive ulnar variance corrected with ulnar shortening osteotomy in this series was 12.5 mm. Hove and Engesaeter [2] published a series of 6 patients with posttraumatic distal radius growth arrest. Three of these patients had ulnar shortening osteotomy correcting a 4 mm positive variance in one and 8 mm in the other two. Lee et al. [3] described ulnar shortening in 3 patients of their series of 10 patients with distal radius growth arrest. The largest correction they performed was 7 mm. Aminian and Schoenecker [4] reported 2 cases requiring ulnar shortening with 6 mm being the largest correction performed. To the best of our knowledge, an acute ulnar shortening of 15 mm is the largest reported.

External fixators and Taylor spatial frames are more frequently used on the radius for deformity and length correction [5, 6]. They are seldom used on the ulna, either as a temporising measure or definitive treatment. In our patient, a mini fixator (AO distraction/compression device) was applied with 2 mm Kirschner wires on either side of the osteotomy site. This provided both alignment control as well as controlled approximation of the bone ends while definitive fixation with a plate was performed. A shortening of 15 mm requires significant force to reduce, maintain, and compress the osteotomy site as illustrated by the deformation of the Kirschner wires (Figure 2(b)). 

In summary, ulnar shortening osteotomy is a suitable procedure for distal radial growth arrest in patients with little or no growth potential. The use of the AO mini distractor intraoperatively facilitated 15 mm of acute shortening of the ulna in a safe and controlled manner affording a good outcome and restoration of grip strength.
